# Indocyanine Green (ICG)-Guided Identification of Hypermetabolic Pancreatic Nodules in Focal Congenital Hyperinsulinism: A Case Report in a 3-Month-Old Infant

**DOI:** 10.1055/s-0042-1742780

**Published:** 2022-02-08

**Authors:** Carlos Delgado-Miguel, Antonio Muñoz-Serrano, Lucas Moratilla, María del Carmen Sarmiento, Miriam Miguel-Ferrero, Nuria Leal, Saturnino Barrena, Leopoldo Martínez

**Affiliations:** 1 Department of Pediatric Surgery, La Paz Children's University Hospital, Madrid, Spain; 2 Institute for Biomedical Research La Paz (IdiPaz), Network for Maternal and Children Health (SAMID), La Paz Children's Hospital, Madrid, Spain

**Keywords:** congenital hyperinsulinism, indocyanine green, laparoscopy, children

## Abstract

Indocyanine green (ICG)-guided near-infrared fluorescence has been recently adopted in pediatric surgery, although its use in the treatment of congenital hyperinsulinism has not been reported. We present a case of focal congenital hyperinsulinism in which ICG-navigation with ICG was used during surgical treatment. A 3-month-old infant was referred to our institution from a peripheral hospital for episodes of persistent hypoglycemia since birth, with no response to intravenous treatment with diazoxide, octreotide, or hydrochlorothiazide. An abdominal positron emission tomography-computed tomography scan showed a hypermetabolic nodule in the proximal portion of the body of the pancreas, compatible with focal congenital hyperinsulinism. A heterozygous mutation in the ABCC gene (Ala1516Glyfs*19) frameshift type inherited from the father was identified, which supported this diagnosis. Laparoscopy-assisted surgery was performed with ICG-guided near-infrared fluorescence, with intravenous injection of 16 mg ICG (2 mg/mg), which allowed localization of the focal lesion in the body of the pancreas. The lesion was resected with bipolar electrocautery and intraoperative histological study confirmed complete resection. Plasma glucose values normalized 6 hours after surgery and the patient was discharged 5 days later. In conclusion, the use of ICG in the treatment of congenital hyperinsulinism helps to identify hypermetabolic pancreatic nodules, decreasing the likelihood of incomplete resection.

## Introduction

In the last few years, the recent advent of fluorescence-guided surgery has changed the intraoperative decision process, essentially through the use of indocyanine green (ICG). 1 As a near-infrared imaging agent, ICG can be traced real time in high resolution, is cost-effective, and is broadly aplicable. 2 It has shown great potential to improve surgical outcomes thanks to its ability to distinguish bile, blood, and lymphatic vessels and disease from nondiseased tissues. 3

Although most of the current applications of ICG are mainly reported for adult surgery, there has recently been an exponential increase in the number of publications in pediatric surgery. 4 In children, its usefulness has been described mainly in hepatobiliary surgery and urology. 5 Recently, its role in the identification of lymphatic, peritoneal and pulmonary metastases in oncological surgeries has also been reported. 6 However, its usefulness in the treatment of congenital hyperinsulinism has not been described to date. We herein report the first application of ICG navigation surgery for the surgical treatment of focal congenital hyperinsulinism by partial pancreatectomy.

## Case Presentation

A 3-month-old infant was referred to our institution from a peripheral hospital for episodes of persistent hypoglycemia since birth. Pregnancy underwent an appropriate prenatal care and an unremarkable prenatal course, with an emergency cesarean delivery at 40 + 2 weeks due to fetal tachycardia. Since birth he presented persistent hypoglycemia in the first hours of life, confirmed by several blood glucose determinations, with need for continuous enteral nutrition by nasogastric tube from the second day of life, with no response to intravenous treatment with diazoxide, octreotide, or hydrochlorothiazide.

**Fig. 1:**
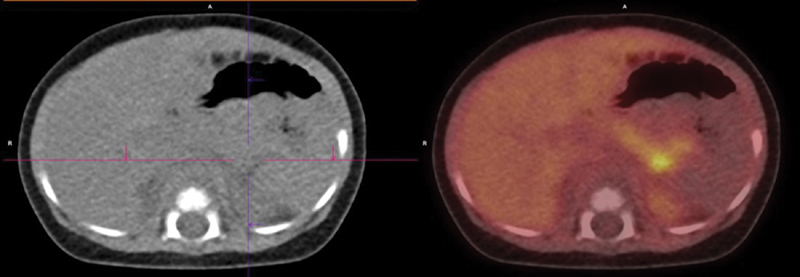
Abdominal 18F-fluoro-L-dihydroxyphenylalanine-positron emission tomography/computed tomography scan showing a hypermetabolic nodule in the distal portion of the body of the pancreas.

On admission, the patient weighed 8.2 kg, and required continuous glucose supplementation via nasogastric tube (17 mL/h) to avoid hypoglycemia. Physical examination revealed a macrosomic appearance with mild axial hypotonia. The abdomen was soft and depressible, with no palpable masses and no other findings of interest. An abdominal 18F-fluoro-L-dihydroxyphenylalanine (18F-DOPA)-positron emission tomography/computed tomography scan showed a hypermetabolic nodule in the distal portion of the body of the pancreas, compatible with focal congenital hyperinsulinism ( Fig. 1 ). A genetic study was performed in both the patients and his parents, which identified a heterozygous mutation in the ABCC gene (Ala1516Glyfs*19) frameshift type inherited from the father, which supported this diagnosis.

**Fig. 2:**
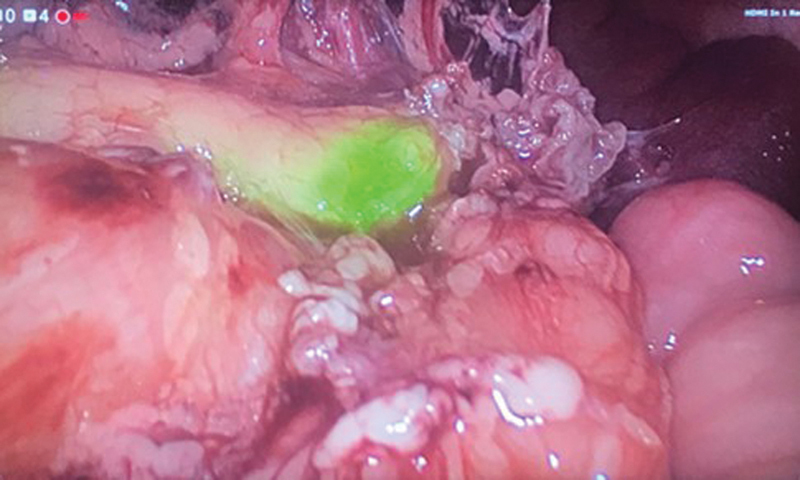
Laparoscopic view of the surgical field, showing a nodule in the distal body of the pancreas with increased uptake of indocyanine green.

Laparoscopy-assisted surgery was performed with ICG-guided near-infrared fluorescence, with intraoperative intravenous injection of 16 mg ICG (2 mg/kg) once the pancreas was identified, without need of injecting it prior to surgery, which allowed localization of the focal lesion in the body of the pancreas ( Fig. 2 ). A laparoscopic system (Stryker Endoscopy, San José, California, United States) was used. The imaging was generated by the high-end full high-definition camera system (1688 4K Inline) connected to a 30-degree optic with a specific filter for optimal detection of the near-infrared fluorescence and standard white light imaging (SPY PHI fluorescence imaging technology). The lesion was resected with bipolar electrocautery and an intraoperative histological study was performed, which confirmed the presence of abundant hyperfunctioning neuroendocrine tissue, with healthy pancreatic tissue surrounding it. After histological confirmation of complete resection of the lesion, a drain was placed in the surgical site (Jackson-Pratt drain). Plasma glucose values normalized 6 hours after surgery, without requiring continuous glucose supplementation. Patient was discharged 5 days later, after removal of the drain, and without postoperative complications.

## Discussion

Congenital hyperinsulinism (CHI) is the most common cause of persistent hypoglycemia in neonates and can lead to irreversible brain damage. 7 Histologically, CHI is classified into three subgroups: diffuse, focal, and atypical forms. 8 Diffuse disease affects all the islets in the pancreas, whereas in focal disease the abnormality is confined to a small region of the pancreas. These two forms of HI are predominantly caused by inactivating mutations of ABCC8 or KCNJ11, the two genes that encode the β-cell ATP-dependent potassium channel. Biallelic recessive, or less commonly, dominant mutations cause diffuse CHI, whereas loss of heterozygosity together with inheritance of a paternal recessive mutation causes focal CHI. 9 Although all the histological subtypes are clinically and biochemically indistinguishable, their differentiation at the histological level is important from the point of the view of management. Patients with diffuse disease on this genetic basis often require near-total pancreatectomy, which has the long-term risk of diabetes mellitus. 10 Conversely, babies with focal disease can be cured with a selective partial pancreatectomy with little risk of subsequent diabetes. 11 The 18-fluoro-DOPA positron emission tomography-computed tomography imaging study is the “gold standard” to make the diagnosis, can help to localize focal lesions, and permit partial pancreatectomy with cure in almost all focal CHI patients. 12

The main difficulty in the treatment of focal CHI is the intraoperative location of the lesion. Focal lesions often have subtle differences in appearance, ranging from a slightly reddish color to a marble-like appearance, and are often slightly firmer compared with normal tissue. Intraoperative high-resolution ultrasound can sometimes help in localizing a focal lesion particularly if the lesion has a pseudocapsule, but most focal lesions have similar echogenicity to normal pancreas. 11 If no focal lesion is identified, several biopsies should be taken sharply with tenotomy scissors from the pancreatic head, body, and tail for intraoperative frozen section analysis. 8 Repeated biopsies increase the risk of complications, such as pancreatic duct injury. In addition, if focal lesions are buried within the pancreatic tissue, it will be impossible to see or feel, so it is necessary to take additional biopsies of suspicious areas for frozen section analysis until the lesion is found. This may increase the risk of developing pancreatic exocrine insufficiency and diabetes which requires lifelong pancreatic enzyme replacement and insulin therapy. 12 The success of surgical treatment in focal CHI consists in resecting the totality of hyperfunctioning cells to avoid recurrence. In this case, ICG-navigation allowed locating the pancreatic hyperfunctioning lesion, and after its resection, intraoperative histological study confirmed the presence of pancreatic hyperfunctioning cells surrounded by healthy pancreatic tissue, so it was considered as a complete resection, with no affected resection margins. If hyperfunctioning cells were observed at the margins of the resected tissue, the extent of the resection should be extended.

Fluorescence-guided surgery with ICG allows precise localization of the focal CHI lesion, helping to identify it intraoperatively and to determine the margin of resection to perform a limited resection, avoiding repeated biopsies. ICG is a water-soluble tricarbocyanine dye that produces fluorescence when excited using near-infrared light with a specific wavelength light (∼820 nm), and it is visualized using specific cameras to be transformed and displayed as visible light. 13 ICG is injected intravenously and due to its protein-binding characteristic, majority of the plasma protein-bound ICG stays within the intravascular space, which allows the identification of structures with increased vascular supply, as in this case, hypermetabolic pancreatic nodules. ICG is then taken up from the plasma almost exclusively by the hepatic parenchymal cells and is entirely secreted by the liver into the bile, with no significant extrahepatic or enterohepatic circulation. The standard dose of 2 mg/kg is much lower than the toxicity level (80 mg/kg), and is considered basically nontoxic, with the exception of iodide allergy which is uncommon. 1 No other adverse effects have been described in relation to the administration of ICG.

To the best of our knowledge, we present the first case of focal CHI in which ICG-navigation was used during surgical treatment. In our experience, ICG technology can facilitate the identification of hypermetabolic nodules in focal CHI, decreasing the likelihood of incomplete resection. It is very useful for safe dissection of hyperfunctioning pancreatic masses because fluorescence allows a more precise identification of the resection margins as well as the pancreatic vascular anatomy and its vascular relationships with other main vessels. To perform this procedure, it is essential to have equipment with compatible technology, which is the main limitation of this technique.

## Notes

New Insights & the Importance for the Pediatric Surgeon Indocyanine green technology can facilitate the identification of hypermetabolic nodules in focal congenital hyperinsulinism, decreasing the likelihood of incomplete resection. It is very useful for safe dissection of hyperfunctioning pancreatic masses because fluorescence allows a more precise identification of the resection margins as well as the pancreatic vascular anatomy and its vascular relationships with other main vessels.
